# Progranulin attenuates liver fibrosis by downregulating the inflammatory response

**DOI:** 10.1038/s41419-019-1994-2

**Published:** 2019-10-07

**Authors:** Wonbeak Yoo, Jaemin Lee, Kyung Hee Noh, Sangmin Lee, Dana Jung, Mohammad Humayun Kabir, Dongmin Park, Cheolju Lee, Ki-Sun Kwon, Ji-Su Kim, Seokho Kim

**Affiliations:** 1 Environmental Disease Research Center, Korea Research Institute of Bioscience and Biotechnology, Daejeon, 34141 Republic of Korea; 2 Industrial Bio-Materials Research Center, Korea Research Institute of Bioscience and Biotechnology, Daejeon, 34141 Republic of Korea; 3 Korea Research Institute of Bioscience and Biotechnology, Daejeon, 34141 Republic of Korea; 4 Center for Theragnosis, Korea Institute of Science and Technology, Seoul, 02792 Korea; 5 Aging Research Center, Korea Research Institute of Bioscience and Biotechnology, Daejeon, 34141 Korea; 6 National Primate Resources Center, Korea Research Institute of Bioscience and Biotechnology, Jeonbuk, 56212 Republic of Korea; 7 Department of Medicinal Biotechnology, College of Health Sciences, Dong-A University, Busan, 49315 Republic of Korea; 8 Present Address: Incepta Vaccine Limited, Dhamrai, Kalampur, Dhaka 1351 Bangladesh

**Keywords:** Mechanisms of disease, Mechanisms of disease, Mechanisms of disease, Mechanisms of disease, Liver fibrosis

## Abstract

Progranulin (PGRN) is a cysteine-rich secreted protein expressed in endothelial cells, immune cells, neurons, and adipocytes. It was first identified for its growth factor-like properties, being implicated in tissue remodeling, development, inflammation, and protein homeostasis. However, these findings are controversial, and the role of PGRN in liver disease remains unknown. In the current study, we examined the effect of PGRN in two different models of chronic liver disease, methionine‐choline‐deficient diet (MCD)-induced non-alcoholic steatohepatitis (NASH) and carbon tetrachloride (CCl4)-induced liver fibrosis. To induce long-term expression of PGRN, PGRN-expressing adenovirus was delivered via injection into the tibialis anterior. In the CCl4-induced fibrosis model, PGRN showed protective effects against hepatic injury, inflammation, and fibrosis via inhibition of nuclear transcription factor kappa B (NF-κB) phosphorylation. PGRN also decreased lipid accumulation and inhibited pro-inflammatory cytokine production and fibrosis in the MCD-induced NASH model. In vitro treatment of primary macrophages and Raw 264.7 cells with conditioned media from hepatocytes pre-treated with PGRN prior to stimulation with tumor necrosis factor (TNF)-α or palmitate decreased their expression of pro-inflammatory genes. Furthermore, PGRN suppressed inflammatory and fibrotic gene expression in a cell culture model of hepatocyte injury and primary stellate cell activation. These observations increase our understanding of the role of PGRN in liver injury and suggest PGRN delivery as a potential therapeutic strategy in chronic inflammatory liver disease.

## Introduction

Liver fibrosis is a response to liver injury due to a variety of causes, including hepatitis infection, excess alcohol consumption, and metabolic disorders. When the injury is sustained, damaged hepatocytes release factors that activate hepatic stellate cells, resulting in excessive extracellular matrix (ECM) production by hepatic stellate cells and non-parenchymal cells^1,2^. This process may result in cirrhosis, the late stage of progressive fibrosis, which is associated with poor long-term outcomes. Despite decades of efforts by clinical and basic researchers, there is no effective treatment for liver fibrosis. Therefore, gaining a greater understanding of the mechanisms underlying liver fibrosis will be required to develop novel therapies that prevent or delay its progression.

Progranulin (PGRN), which is characterized by repeats of a cysteine-rich granulin motif, is a secretory glycoprotein expressed by a wide variety of cell types in the blood and cerebrospinal fluid^3^. Recently, PGRN has been shown to have pleiotropic effects in tissue development, regeneration, inflammation, metabolic disease, and neurodegeneration^4–7^. PGRN overexpression has been found in many types of cancer, and was found to promote tumor growth under pathological conditions^8–10^. However, PGRN has a protective effect in neurogenerative disease^11,12^, while PGRN deficiency inhibits insulin resistance and metabolic dysfunction^13,14^. Although PGRN is dysregulated in different diseases, its exact role in disease progression remains controversial. Interestingly, PGRN has been reported to have anti-inflammatory effects, as it directly binds to tumor necrosis factor receptor-1 (TNFR1) and TNFR2^4,15–18^. The effect of PGRN on liver fibrosis has not been investigated, but based on these findings, it is conceivable that PGRN might inhibit inflammation during chronic liver disease by modulating tumor necrosis factor-α (TNF-α) signaling.

The present study aimed to expand our knowledge of the role of PGRN in the progression of hepatic fibrosis. We demonstrate that PGRN administration protects against liver fibrosis and non-alcoholic steatohepatitis by reducing macrophage activation and collagen accumulation. These findings provide new insights into the pathogenesis of liver fibrosis that could lead to the identification of better therapeutic targets.

## Results

### Progranulin reduced liver fibrosis after CCl4-induced chronic liver injury

**Fig. 1:**
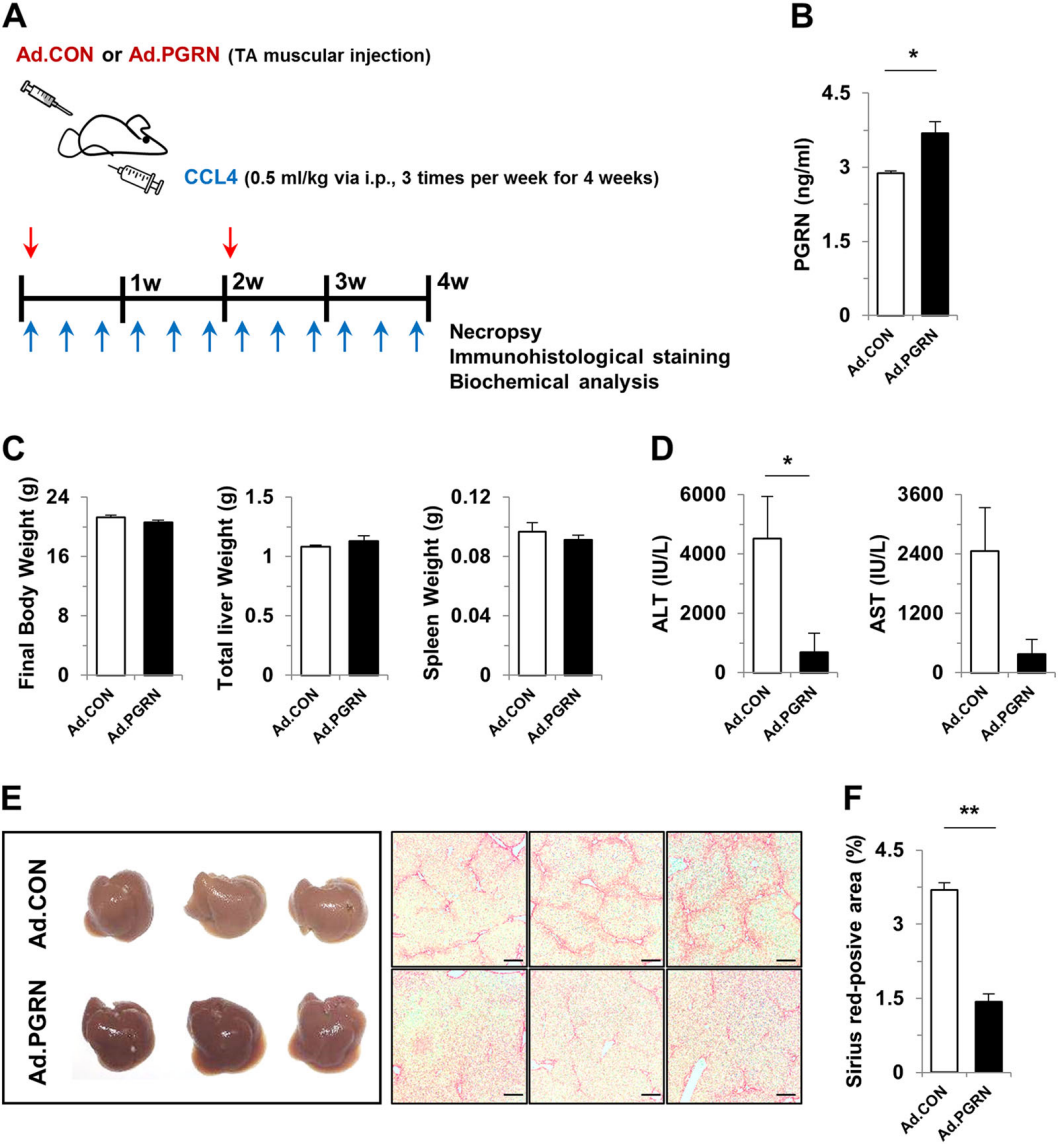
PGRN attenuates CCl4-induced fibrosis in mice. **a** Schematic diagram of the study. Liver fibrosis was induced by CCl4 injection for 4 weeks. Ad.CON, control adenovirus + CCl4; Ad.PGRN, PGRN adenovirus + CCl4; TA, tibialis anterior. **b** Serum PGRN levels. **c** Final body, liver, and spleen weights of Ad.CON and Ad.PGRN mice. **d** Serum levels of ALT and AST. **e** Liver morphology and staining with Sirius Red. Scale bar: 200 μm. **f** Quantification of the Sirius Red-positive area per high-powered field. Graphs show the mean ± SEM. *p < 0.05 and **p < 0.01 versus the corresponding control, as shown

To investigate the role of PGRN in the pathogenesis of liver fibrosis, liver injury was induced by repeated intraperitoneal injection of CCl4, and wild-type mice were administered a control or PGRN-expressing adenovirus for 4 weeks (Fig. 1a). Serum levels of PGRN were significantly increased in PGRN mice (Fig. 1b). Administration of PGRN significantly decreased serum levels of AST and ALT compared with control mice, although the body, liver, and spleen weights were not significantly different between the two groups (Fig. 1c, d). On gross observation, the livers of PGRN-treated mice were more reddish in color than those of control mice. Moreover, collagen deposition was markedly reduced in liver sections of PGRN-treated mice compared with control mice, as shown by Sirius Red staining (Fig. 1e, f). Additionally, there were no differences in the tibialis anterior (TA) or gastrocnemius (GA) muscles between mice treated with control or PGRN-expressing adenovirus by intramuscular injection (Supplementary Fig. 1). These data suggest that intramuscular delivery of PGRN attenuates CCl4-mediated hepatic fibrosis and injury.

**Fig. 2:**
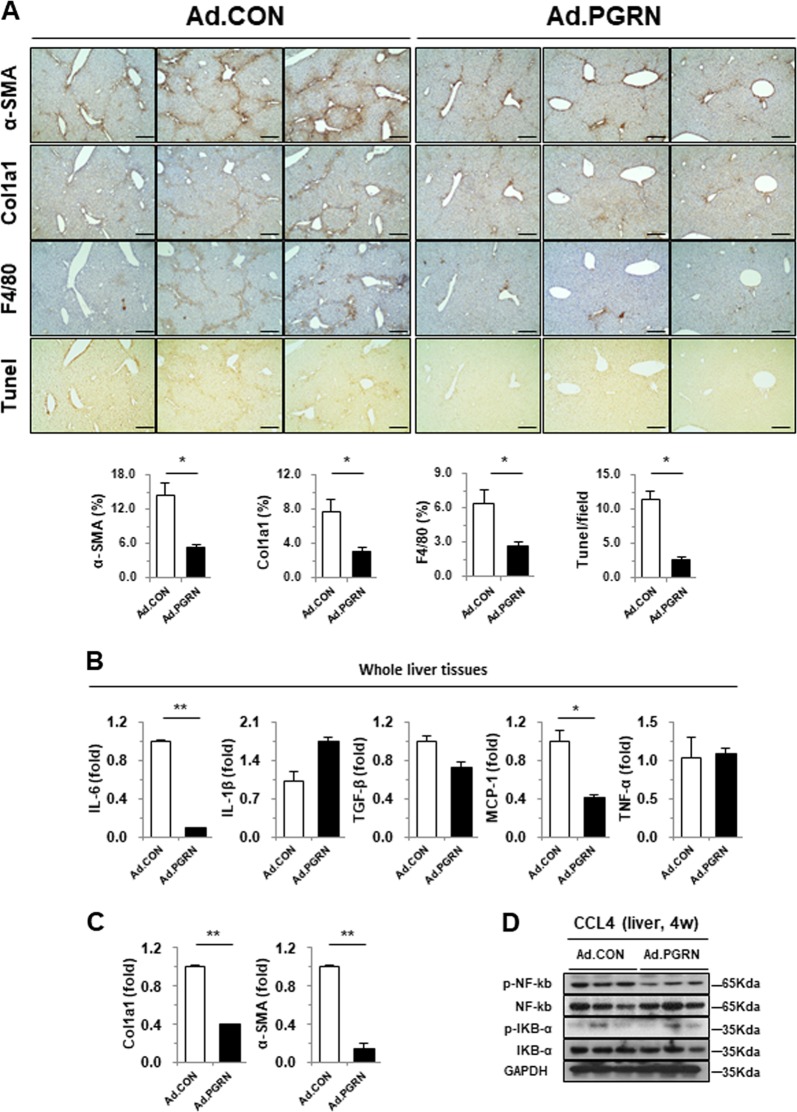
Effect of PGRN on liver fibrosis-related protein expression in CCl4-treated mice. **a** Immunohistochemical staining of liver sections for α-SMA, collagen 1a1, F4/80 antigen, and apoptotic cells (TUNEL). The mean number of positively stained cells per high-powered field was quantified. Scale bar: 200 μm. **b**, **c** Whole liver tissues were subjected to gene expression analysis by real-time qPCR. **d** Liver tissues were subjected to western blot analysis. Graphs show the mean ± SEM. *p < 0.05 and **p < 0.01 versus the corresponding control, as shown

To further address the relationship between PGRN and hepatic inflammation and fibrosis, immunohistochemical staining was performed on serial liver sections. As expected, less liver fibrosis and injury was observed in PGRN-treated mice, as assessed by the expression of α-SMA and collagen, as well as by TUNEL staining. In addition, the number of F4/80 antigen-positive macrophages, which play a pivotal role in hepatic fibrogenesis, was reduced in PGRN-treated mice compared with control mice (Fig. 2a). Consistent with the histology analysis, the expression of α-SMA and Col1a1 was significantly decreased in the livers of PGRN-treated mice compared with controls (Fig. 2b, c). Inflammation is also associated with CCl4-induced liver injury, and activation of NF-κB is commonly used as an inflammatory marker. Immunoblot analyses revealed that NF-κB phosphorylation was significantly reduced in PGRN-treated livers compared with control livers (Fig. 2d). Collectively, these data suggest that PGRN ameliorates hepatic fibrosis in the CCl4-induced liver injury model by inhibiting inflammation.

### Progranulin attenuates cellular inflammation and fibrogenesis in vitro

**Fig. 3:**
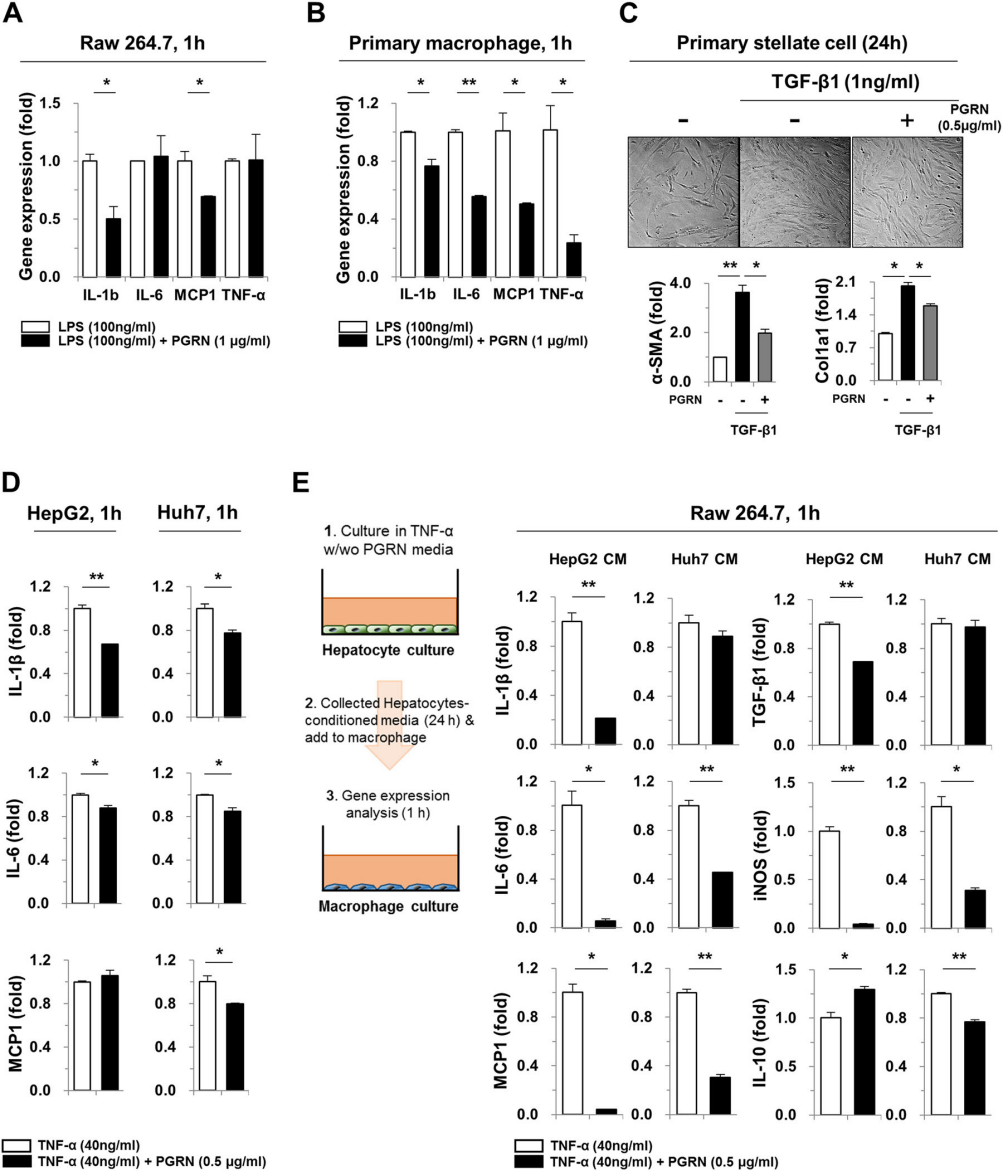
The effect of PGRN on the macrophage inflammatory response to LPS. **a**, **b** Raw 264.7 cells and primary macrophages were pre-treated with PGRN for 30 min. They were then treated with LPS for an additional hour, and the expression of inflammatory genes was analyzed by real-time qPCR. **c** Human primary stellate cells were pre-treated with PGRN for 30 min. They were then treated with TGF-β1 for an additional 24 h, and gene expression was analyzed. **d** HepG2 and Huh7 cells were pre-treated with PGRN for 30 min and then stimulated with TNF-α for an additional 24 h and subjected to real-time qPCR analysis. **e** Raw 264.7 cells were treated with conditioned media from HepG2 or Huh7 cells treated with TNF-α for 24 h with or without 30 min of PGRN pre-treatment. After 1 h, the Raw 264.7 cells were subjected to real-time qPCR analysis. Graphs show the mean ± SEM. *p < 0.05 and **p < 0.01 versus the corresponding control, as shown

To investigate the effect of PGRN on the inflammatory response in more detail, LPS, TGF-β1, and TNF-α were used to induce macrophage activation, stellate cell activation, and hepatic injury, respectively. As shown in Fig. 3a, IL-1β and monocyte chemoattractant protein-1 (MCP-1) expression was decreased significantly in Raw 264.7 cells pre-treated with PGRN prior to stimulation with LPS, compared with cells treated with LPS only. Similarly, pre-treatment with PGRN decreased the expression of IL-1β, IL-6, MCP-1, and TNF-α in LPS-treated primary macrophages (Fig. 3b). To examine the role of PGRN in stellate cell activation, human primary stellate cells were activated with TGF-β1 with or without PGRN pre-treatment. At 24 h, the expression of α‐SMA and Col1a1 was significantly reduced in PGRN-pre-treated cells (Fig. 3c). In addition, HepG2 and Huh7 cells were stimulated with TNF-α for the indicated times, and the expression of pro-inflammatory cytokines was evaluated. IL-1β and IL-6 were modestly decreased in PGRN pre-treated cells compared with cells activated with TNF-α without PGRN pre-treatment (Fig. 3d). Next, we examined whether PGRN could inhibit the ability of injured hepatic cells to activate macrophages. Conditioned media from PGRN pre-treated, TNF-α-stimulated HepG2 and Huh7 cells decreased the expression of pro-inflammatory cytokines, including IL-1β, IL-6, MCP-1, and TGF-β1, by macrophages (Fig. 3e). Interestingly, decreased expression of iNOS, a marker of M1 macrophage polarization, was also observed. Collectively, these data suggest that PGRN not only reduces hepatic injury, but also decreases macrophage infiltration and activation, as well as stellate cell activation.

### Progranulin reduced hepatic steatosis and injury in a diet-induced NASH model

**Fig. 4:**
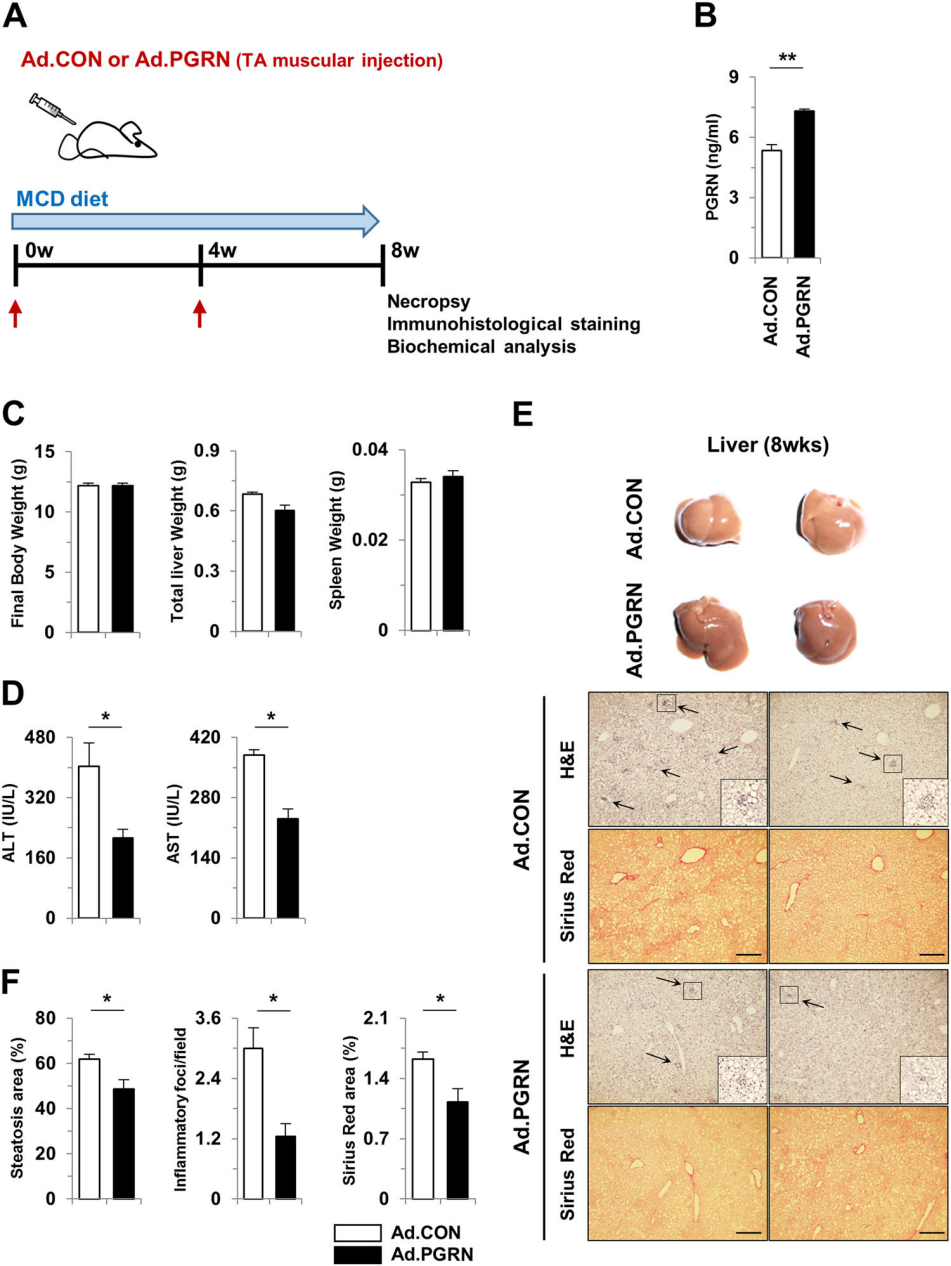
PGRN attenuates steatohepatitis induced by a MCD diet in mice. **a** Schematic diagram of the animal study. NASH was induced with a MCD diet for 8 weeks. Ad.CON, control adenovirus + MCD diet; Ad.PGRN, PGRN-expressing adenovirus + MCD diet; TA, tibialis anterior. **b** Serum PGRN levels. **c** Final body, liver, and spleen weights of Ad.CON and Ad.PGRN mice. **d** Serum ALT and AST levels. **e** Liver sections were stained with H&E and Sirius Red. Scale bar: 200 μm. **f** Quantification of steatosis, inflammatory foci, and Sirius Red staining. Graphs show the mean ± SEM. *p < 0.05 and **p < 0.01 versus the corresponding control, as shown

**Fig. 5:**
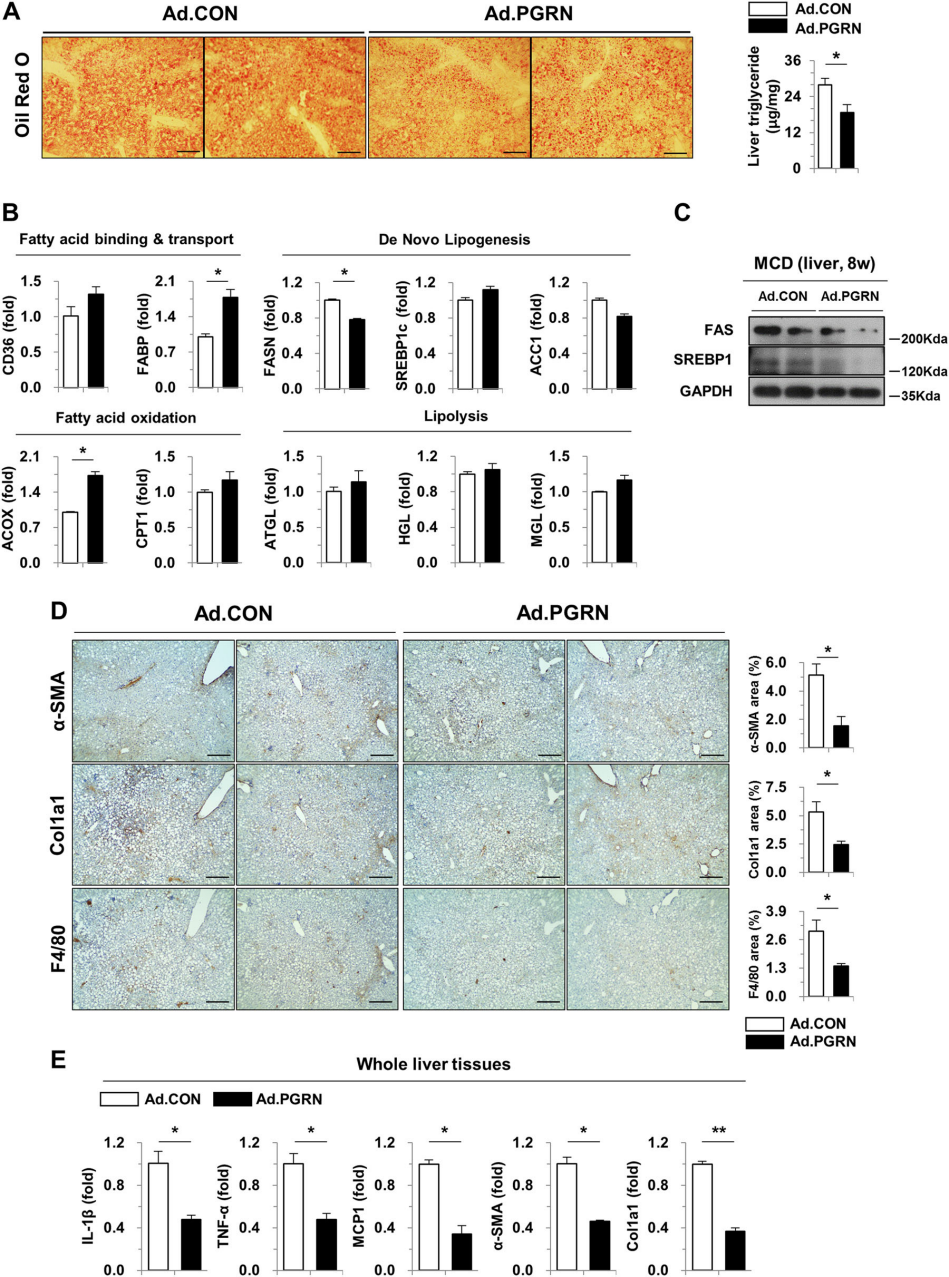
PGRN treatment decreases MCD diet-induced hepatic steatosis and fibrosis in mice. **a** Oil Red O staining and liver TG content. Scale bar: 200 μm. **b** Liver expression of genes involved in lipid metabolism was determined by real-time qPCR. **c** Liver tissues were subjected to western blot analysis. **d** Immunohistochemical staining for α-SMA, collagen 1a1, and F4/80 antigen. Scale bar: 200 μm. **e** Whole liver tissues were subjected to gene expression analysis by real-time qPCR. Graphs show the mean ± SEM. *p < 0.05 and **p < 0.01 versus the corresponding controls, as shown

Next, to extend our previous findings in a CCl4-induced liver injury model, we used a well-established mouse model of MCD diet-induced NASH (Fig. 4a). Serum levels of PGRN were significantly increased in PGRN-treated mice (Fig. 4b). As shown in Fig. 4c, d, PGRN-treated mice showed significantly decreased serum AST and ALT levels after 8 weeks of a MCD diet, with no difference in body, liver, or spleen weights. On gross observation, the livers of PGRN mice were less yellow in color than those of control mice (Fig. 4e). Notably, liver steatosis, inflammation, and collagen accumulation were significantly attenuated by PGRN in MCD diet-induced NASH (Fig. 4f). Consistent with the observation that PGRN-treated mice had less liver fat than control mice, as reflected by TG levels (Fig. 5a), the expression of fatty acid synthase (FAS) was reduced in PGRN-treated mice, while the level of acyl-CoA oxidase 1 (ACOX) was significantly increased, indicating reduced hepatic lipid accumulation (Fig. 5b). Moreover, immunoblot analysis of liver tissues revealed that expression of FAS and sterol regulatory element-binding protein 1 (SREBP1) was significantly reduced in PGRN-treated mice (Fig. 5c). PGRN mice fed a MCD diet exhibited less liver fibrosis and reduced numbers of infiltrating hepatic macrophages, as shown by Sirius Red staining and immunohistochemical staining for α-SMA, collagen, and F4/80 antigen (Fig. 5d). Consistent with these findings, the expression of IL-1β, TNF-α, MCP-1, α-SMA, and Col1a1 was significantly reduced in whole liver tissues of PGRN-treated mice compared with control mice (Fig. 5e). Together, these findings demonstrate that PGRN reduces steatohepatitis and liver fibrosis in a mouse NASH model.

### Progranulin reduced inflammatory gene expression in PA-induced macrophages

**Fig. 6:**
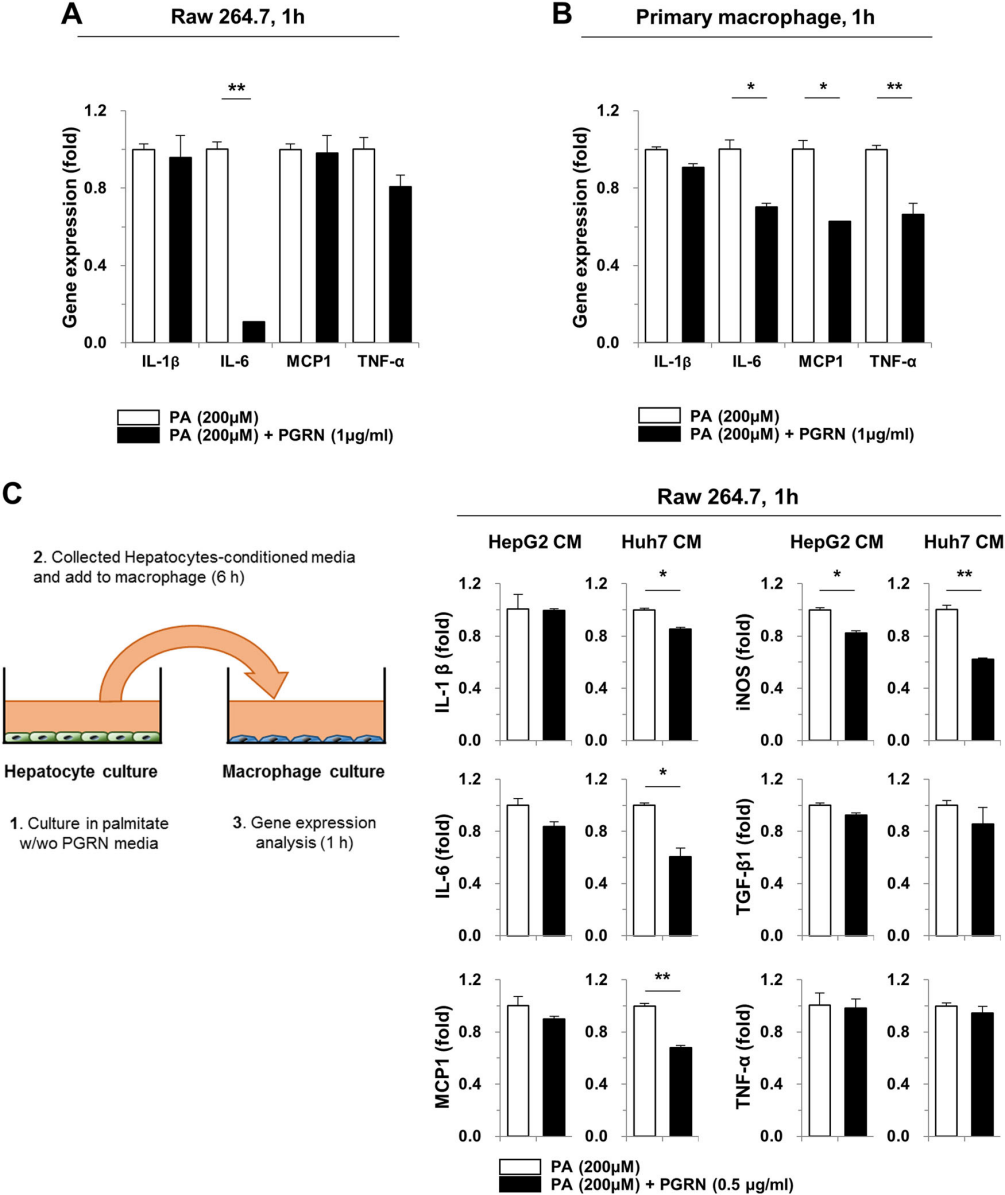
The effect of PGRN on the macrophage response to palmitate. **a**, **b** Raw 264.7 cells and primary macrophages were pre-treated with PGRN for 30 min. They were then treated with palmitate for an additional hour, and expression of inflammatory genes was analyzed by real-time qPCR. **c** Raw 264.7 cells were treated with conditioned media from HepG2 or Huh7 cells treated with palmitate for 6 h with or without 30 min of PGRN pre-treatment. Raw 264.7 cells were exposed to the conditioned media for 1 h and then subjected to real-time qPCR analysis. Graphs show the mean ± SEM. *p < 0.05 and **p < 0.01 versus the corresponding control, as shown

Excessive hepatic lipid accumulation can result in steatosis and lead to hepatic inflammation and fibrosis. Importantly, free fatty acids can induce pro-inflammatory cytokine expression in macrophages and hepatocytes. Since hepatic inflammation was strongly reduced in PGRN-treated mice with MCD diet-induced NASH, our next objective was to define the role of PGRN in the response to palmitate treatment in vitro. Palmitate treatment of Raw 264.7 macrophages increased IL-6 expression, which was abrogated by PGRN pre-treatment (Fig. 6a). Additionally, pre-treatment with PGRN downregulated the expression of pro-fibrogenic genes in primary macrophages, including IL-6, MCP-1, and TNF-α, compared with treatment with palmitate alone (Fig. 6b). These data suggest that PGRN reduced hepatic inflammation in the CCl4-induced fibrosis model by altering macrophage activation. Based on these findings, we further asked whether palmitate‐induced hepatic injury can affect macrophage activation, similarly to CCl4. HepG2 and Huh7 cells were treated with palmitate in vitro for 6 h with or without PGRN pre-treatment, and conditioned media was collected (Fig. 6c). Treatment of Raw 264.7 cells with conditioned media from PGRN-pre-treated cells reduced their expression of IL-1β, IL-6, MCP-1, and iNOS compared with treatment with conditioned media from cells stimulated with palmitate without PGRN pre-treatment. These data strongly suggest that the anti-inflammatory effect of PGRN on hepatic macrophages protects mice from hepatic injury induced by a MCD diet. To confirm this, we next investigated the role of PGRN in palmitate-treated hepatic cells. In this context, no significant effect of PGRN was seen on palmitate-induced hepatic lipid accumulation or related gene expression (Supplementary Fig. 2a, b), although the expression of IL-6 and MCP-1 was reduced slightly in PGRN-treated hepatic cells exposed to palmitate (Supplementary Fig. 2c). These data suggest that PGRN not only decreases hepatic inflammation, but also partially inhibits hepatic steatosis.

## Discussion

In this study, we show that intramuscular administration of PGRN-expressing adenovirus had beneficial effects in two different models of chronic liver disease. In both models, PGRN reduced macrophage infiltration and activation, which resulted in reduced hepatic fibrosis. The exact mechanisms responsible for the amelioration of hepatic inflammation and fibrosis in PGRN-treated mice remain unclear, but the following data are relevant. First, PGRN-treated mice showed less hepatic injury and decreased production of pro-inflammatory cytokines, which are potent activators of the immune system in the liver. Second, the livers of PGRN-treated mice had decreased phosphorylation of NF-κB, which plays a key role in the transcription of diverse pro-inflammatory cytokines and chemokines during hepatic fibrogenesis^19,20^. Importantly, accumulating evidence suggests that PGRN is a ligand for the TNF receptor^15,18,21^.

The increased activation of inflammatory macrophages results in increased production of pro-inflammatory cytokines, which in turn promotes the progression of chronic liver disease^22,23^. Moreover, hepatic macrophages play a central role in the inflammatory response and fibrogenic process during hepatic injury. As illustrated in Figs. 1 and 2, PGRN significantly attenuated CCl4-induced hepatic fibrosis with a concomitant reduction in α-SMA-, Col1a1-, and F4/80 antigen-positive cells. Next, we addressed whether macrophages are affected by hepatic injury and whether this translates to improved macrophage inflammatory responses. Macrophages treated with conditioned media from PGRN-pre-treated injured hepatic cells showed reduced expression of pro-inflammatory cytokines compared with macrophages treated with conditioned media from injured hepatic cells that had not been pre-treated with PGRN. Consistent with the data from the model of CCl4-induced liver injury, PGRN treatment improved liver function, reduced hepatic steatosis, reduced hepatic TG levels, reduced collagen accumulation and infiltration of F4/80 antigen-positive cells in a MCD diet-induced NASH model (Figs. 4 and 5). A previous study demonstrated that TNF-α induces hepatic steatosis^24^. Intraperitoneal injection of TNF-α enhanced hepatic fat deposition and TG content via lipogenic metabolism. In the present study, PGRN treatment attenuated the steatohepatitis induced by MCD diet. These effects of PGRN might be due to its effects on the PGRN/TNF-α balance, as it was previously shown that PGRN competitively binds TNF receptors, potently blocking TNF-TNFR interactions and TNFR downstream signaling^4,18^. Although PGRN pre-treatment did not affect lipid accumulation in a palmitate-induced cell culture model of steatosis, PGRN did show partial anti-inflammatory effects. PGRN also exhibited anti-inflammatory effects in macrophages, not only by direct treatment, but also indirectly, through its effects on palmitate-treated hepatic cells. Collectively, these results suggest that PGRN can directly and/or indirectly affect hepatocytes, macrophages, and stellate cells.

To further understand the functional relationships of PGRN, we evaluated its protein-protein interaction network using STRING-based network analysis. This network included 30 genes with various molecular functions, including cytochrome-c oxidase (COX) activity and TNFR activity. COX signaling is one of the most important pathogenic events in hepatic oxidative stress. In addition, TNF-related signaling is a major event in hepatic inflammation, inducing cytokine release and immune cell recruitment^25^. Moreover, Kyoto Encyclopedia of Genes and Genomes (KEGG) pathway analysis of PGRN signaling also showed the differential expression of genes involved in non-alcoholic fatty liver disease, adipocytokine signaling, TNF signaling, and oxidative phosphorylation, which suggests a relationship with inflammatory disease. These data suggest that PGRN can function in a variety of biological processes, including immune responses, metabolism, and energy pathways (Supplementary Fig. 3). Further gene expression analysis of the pathways regulated by PGRN will be important in the future.

Although PGRN plays important roles in multiple physiological and pathological conditions, the role of PGRN in metabolism remains controversial. A recent study suggested that PGRN has anti-inflammatory effects in the context of wound repair, autoimmune disease (psoriasis vulgaris), central nerve system damage, arthritis, and acute ischemia-reperfusion injury^5,26–29^, while PGRN dysregulation has been shown to be dysregulated in obesity, insulin resistance, type 2 diabetes mellitus, dyslipidemia, and renal disease^30–33^. Additionally, the serum PGRN level was an independent marker of liver fibrosis and was positively associated with elevated liver enzymes^34,35^. Here, we observed that in vivo administration of PGRN to wild-type mice with CCl4-induced liver injury or MCD diet-induced NASH significantly reduced inflammation and fibrosis, reflecting a beneficial role for PGRN in chronic liver injury. The discrepancies with our results might possibly be related to the balance between progranulin and granulins. Intact progranulin (full-length PGRN) exerts anti-inflammatory effects through the inhibition of TNF-α signaling, while proteolytically processed PGRN (i.e., proteolytically released granulins) may stimulate the production of pro-inflammatory cytokines^36–38^. Thus, the beneficial anti-fibrotic effects of PGRN administration are accompanied by decreased macrophage activation and infiltration of liver tissue, consistent with their well-known role as key regulators of liver fibrogenesis. The differential contribution of the intact and proteolytically cleaved forms of PGRN in liver fibrosis will be further investigated in follow-up studies.

In conclusion, this study showed that, in mouse models of hepatic fibrosis and NASH, PGRN administration improved inflammation and fibrosis and also reduced steatosis and hepatocellular injury, at least in part by altering macrophage activation and infiltration. These findings suggest that PGRN could have efficacy as an anti-fibrotic agent.

## Materials and methods

### Animal care and adenovirus delivery

For the chronic liver injury model, 7-week-old C57BL/6 female mice (Narabio Inc., Seoul, Korea) were injected intraperitoneally with 20% carbon tetrachloride (CCl4) (v/v in olive oil, 0.5 ml/kg body weight, three times per week) to induce hepatic fibrosis, as described by Seo et al.^39^. Alternatively, mice were fed a methionine-choline-deficient (MCD) diet for 8 weeks^40^. Adenoviral particles expressing mouse PGRN (Ad.PGRN, AD-m-GRN) and control particles (Ad.CON, AD-CMV-null) were purchased from VectorBiolabs (Philadelphia, PA, USA). To induce stable expression of PGRN, adenoviral particles were diluted in a physiological saline solution and injected into the tibialis anterior (TA) muscle at a titer of 1 × 10^9^ infectious units/injection, using an insulin syringe. The dose of viral vector was chosen based on previous reports, in order to limit vector-associated toxicity and inflammation^41,42^. All animal housing was in compliance and experiments were conducted in accordance with the Korea Research Institute of Bioscience and Biotechnology (KRIBB) Institutional Animal Care and Use Committee Guidelines.

### mPGRN assay

Blood was collected and centrifuged at 8000 rpm for 20 min at 4 °C. The serum concentration of mPGRN was measured by sandwich enzyme-linked immunosorbent assay (ELISA) using a mouse PGRN ELISA kit (R&D Systems, Minneapolis, MN, USA).

### Biochemical analysis

Serum samples were prepared at the end of each experiment and stored at −70 °C until further analysis. Alanine aminotransferase (ALT) and aspartate aminotransferase (AST) levels were determined with an automated blood chemistry analyzer (Hitachi 7150; Tokyo, Japan). Liver triglycerides (TGs) were measured using an enzymatic assay kit (ab65336; Abcam, Cambridge, UK) following the manufacturer’s instructions.

### Cell culture

Mouse primary peritoneal macrophages were isolated from C57BL/6 female mice by lavage with cold phosphate-buffered saline (PBS). Following harvest, cells were plated for 2 h to facilitate cell attachment. Non-adherent cells were removed by washing with warm PBS, and the adherent cells were incubated in RPMI-1640 (Hyclone, Rockford, IL, USA) supplemented with 10% fetal bovine serum (FBS; Hyclone) and 1% antibiotic-antimycotic solution (AA; Gibco/Thermo Fisher Scientific, Waltham, MA, USA). Human primary stellate cells were purchased from ScienCell Research Laboratories (Carlsbad, CA, USA) and maintained with supplementary materials. HepG2 and Huh7 cells were obtained from the American Type Culture Collection (Manassas, VA, USA) and maintained in Dulbecco’s modified Eagle’s medium (DMEM; Hyclone) containing 10% FBS and 1% AA in 5% CO_2_ at 37 °C. Raw 264.7 cells were cultured in RPMI-1640 containing 10% FBS and 1% AA. Recombinant human PGRN, mouse PGRN, human TNF-α, and transforming growth factor-β1 (TGF-β1) were obtained from R&D Systems. Lipopolysaccharide (LPS) derived from *Escherichia coli* serotype 055:B5 was purchased from Sigma-Aldrich (St. Louis, MO, USA). Palmitate (Sigma-Aldrich) was conjugated with bovine serum albumin (BSA; fatty acid-free; Sigma-Aldrich) as described previously^43^.

### Treatment of Raw 264.7 cells with conditioned media from injured hepatic cells

HepG2 and Huh7 cells were cultured in complete medium (DMEM supplemented with 10% FBS and 1% AA) at 37 °C. After pre-treatment with 0.5 μg/ml PGRN for 30 min, 40 ng/ml TNF-α was added to the cells. After 24 h, the cells were washed twice with PBS, and the media were replaced with complete culture medium (RPMI-1640 containing 10% FBS and 1% AA). After another 24 h, the supernatants were collected and filtered through 0.22 μm filters (Sartorius, Goettingen, Germany) to remove cells and debris. The collected conditioned media were mixed with an equal volume of complete culture medium. The resulting medium was used to treat Raw 264.7 cells. To mimic the conditions of hepatic steatosis with inflammation in cultured hepatic cells, HepG2 and Huh7 cells were pre-treated with 0.5 μg/ml PGRN for 30 min, and then 100 or 200 μM palmitate was added for 24 h. After treatment, the culture media were collected and used to treat Raw 264.7 cells as described above.

### Western blotting

Protein from tissues was extracted with Pro-Orep (iNtRON Bio, Seongnam, Korea) according to the manufacturer’s instructions. Western blot analysis was performed with protein lysates using antibodies to sterol regulatory element-binding protein 1 (SREBP1; Santa Cruz Biotechnology, Santa Cruz, CA, USA) and phospho-NF-κB, total NF-κB, phospho-IκBα, total IκBα, fatty acid synthase (FAS), and GAPDH (all from Cell Signaling Technologies, Danvers, MA, USA).

### RNA isolation and gene expression analysis

Total RNA was extracted from liver tissues and cultured cells with TRIzol (Invitrogen/Thermo Fisher Scientific), according to the manufacturer’s protocol. cDNA was synthesized using the RT Kit (Biofact, Daejeon, Korea). Quantitative real-time PCR (real-time qPCR) was performed with Power SYBR Green PCR master mix (Applied Biosystems/Thermo Fisher Scientific). Values were expressed as the fold change compared with the expression of GAPDH. The primers used are listed in Supplementary Table 1.

### Histological staining

Tissue sections were immunostained with antibodies against α-smooth muscle actin (α-SMA) (M0851; Dako, Glostrup, Denmark), collagen 1a1 (Col1a1) (ab34710; Abcam), and F4/80 antigen (sc-377009; Santa Cruz Biotechnology) using the Dako REAL EnVision Detection System according to the manufacturer’s instructions. For Sirius Red staining, tissues were submerged in Picro Sirius Red Stain (ab150681; Abcam) for 30 min, followed by a 1 min wash in 0.1 N HCl, and the slides were then dehydrated and mounted. To assess the degree of inflammation, the number of inflammatory foci per five fields was quantified from hematoxylin and eosin (H&E)-stained liver sections. Oil Red O staining was performed as previously described^44^. Stained sections were observed and photographed under a light microscope (Olympus Optical Co., Ltd., Tokyo, Japan). All results are from triplicate experiments.

### Terminal deoxynucleotidyl transferase-mediated dUTP-biotin nick-end labeling assay

Apoptotic hepatocytes were labeled in situ using a Terminal deoxynucleotidyl transferase-mediated dUTP-biotin nick-end labeling assay (TUNEL) peroxidase detection kit (DeadEnd Colorimetric TUNEL System; Promega, Madison, WI, USA) according to the manufacturer’s protocol.

### Statistical analysis

Graphing and statistical analysis (Student’s *t*-tests or one-way analyses of variance, for multiple comparisons) were performed using GraphPad Prism 5 (GraphPad Software, La Jolla, CA, USA). The data are presented as the mean ± standard error of the mean (SEM). Differences were considered statistically significant if *p* < 0.05.

## Supplementary information

Supplementary figures

Supplementary table
