# Supplementary material for: Progranulin attenuates liver fibrosis by downregulating the inflammatory response
Source: Cell Death Dis. 2019 Oct 7;10(10):758. doi: 10.1038/s41419-019-1994-2 (PMC6779917; doi:10.1038/s41419-019-1994-2)
Supplement: Supplementary file 1 — Supplementary figures [file 41419_2019_1994_MOESM1_ESM.docx]

**
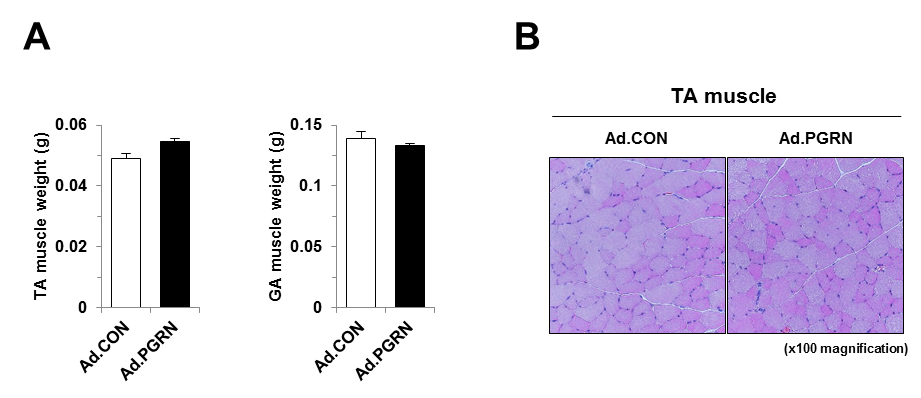
**

**Supplementary Figure 1. Adenoviral delivery of PGRN to TA muscle.** (A) TA and GM Muscle weight of different groups. (B) H&E staining of TA muscle reveals no difference in PGRN virus with CCl4 (left) compared to control virus with CCl4 (right) Scale bar: 200 μm.

**
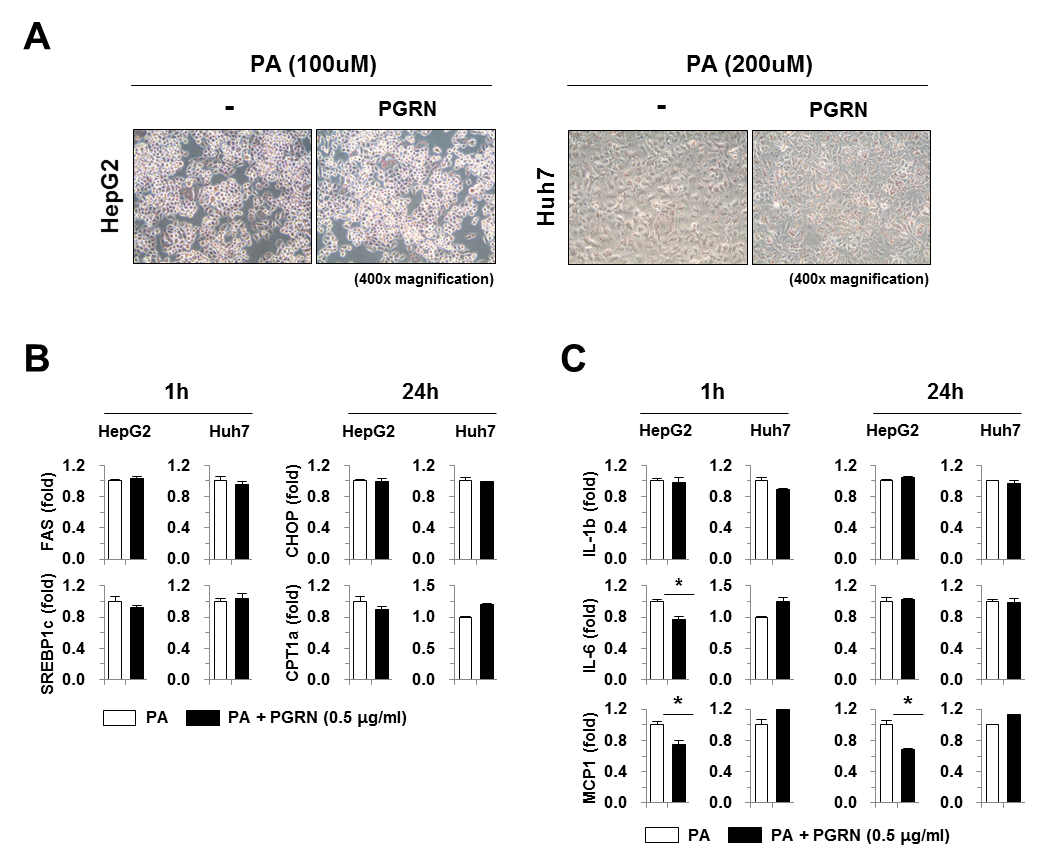
**

**Supplementary Figure 2.** (A) Representative images of HepG2 and Huh7 cells, with or without pretreatment of PGRN for 30 min. These pretreatment were added vehicle or treated with palmitate for 24 h. After 24 h incubation with palmitate, the cells were stained with Oil Red O and gene expression analyzed by qRT-PCR (B and C). Graphs show mean ± SEM, *p < 0.05 versus the corresponding control as shown.

**
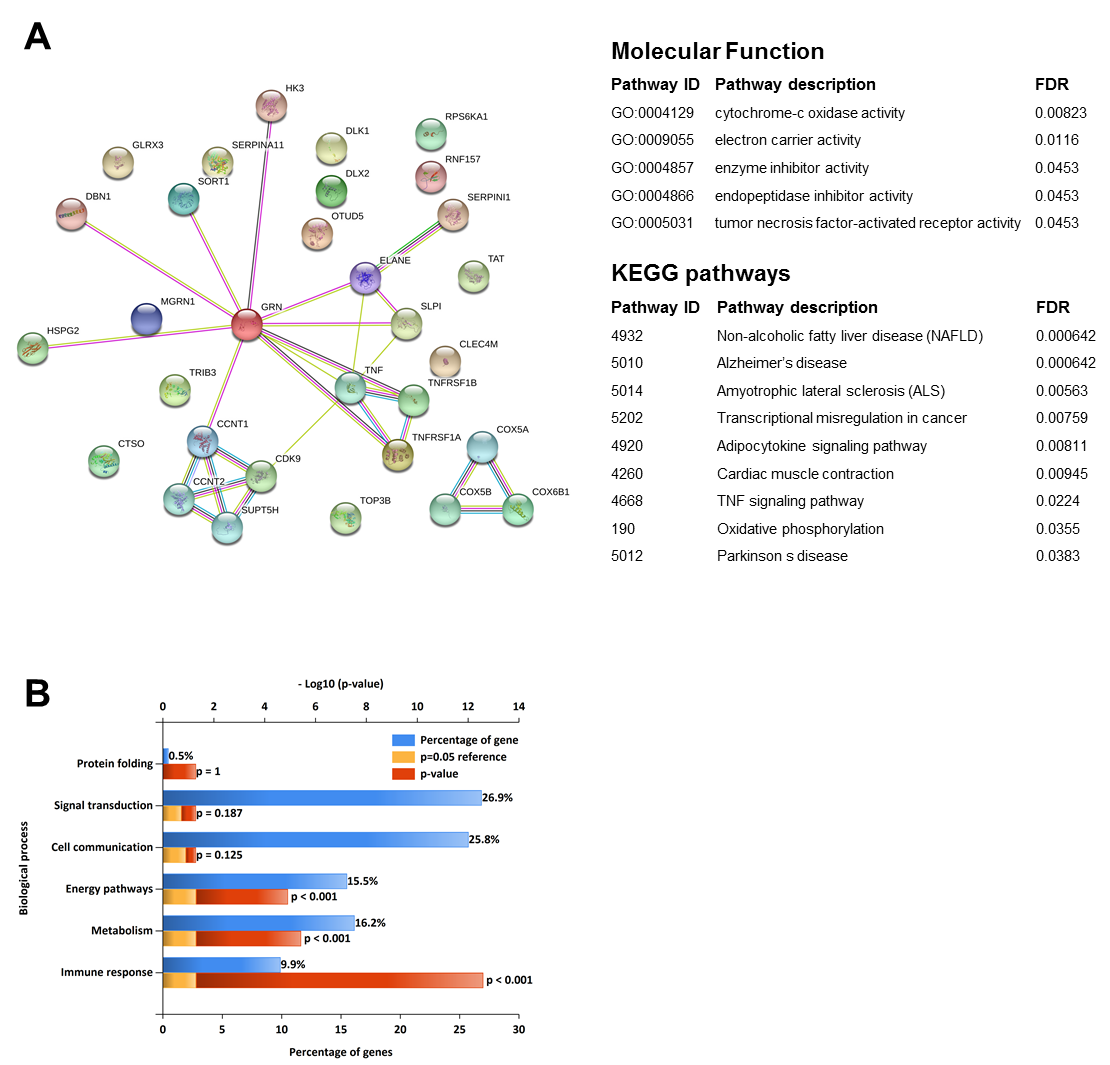
**

**Supplementary Figure 3. PGRN orchestrates inflammation related signaling and immune network signature.** (A) STRING-based network analysis for interaction genes with the PGRN. Functional enrichments of the network with PGRN mainly involved in inflammation related signaling and disease. (B) Biological process analysis of all genes from detected in A. GO was performed using FunRich database. Note a strong contribution of an immune response.
