# Supplementary material for: Progranulin attenuates liver fibrosis by downregulating the inflammatory response
Source: Cell Death Dis. 2019 Oct 7;10(10):758. doi: 10.1038/s41419-019-1994-2 (PMC6779917; doi:10.1038/s41419-019-1994-2)
Supplement: Supplementary file 2 — Supplementary table [file 41419_2019_1994_MOESM2_ESM.docx]

**Supplementary Table 1. Primer information**

| **Gene** | **Forward (5'-3')** | **Reverse (5'-3')** |
| --- | --- | --- |
| **Mouse** |  |  |
| α-SMA | CTGACAGAGGCACCACTGAA | GAAGGAATAGCCACGCTCAG |
| Col1a1 | TCCTCCAGGGATCCAACGA | GGCAGGCGGGAGGTCTT |
| F4/80 | TCAAGGACACGAGGTTGCTGA | CCAAGGGGCCAATCTGGAA |
| IL-6 | TCCATCCAGTTGCCTTCTTG | TTCCACGATTTCCCAGAGAAC |
| IL-1β | GCCCATCCTCTGTGACTCAT | AGGCCACAGGTATTTTGTCG |
| TFG-β | TGGTTGTAGAGGGCAAGGAC | TTGCTTCAGCTCCACAGAGA |
| MCP-1 | TCAGCCAGATGCAGTTAACGC | TCTGGACCCATTCCTTCTTGG |
| TNF-α | AAGCCTGTAGCCCACGTCGTA | AAGGTACAACCCATCGGCTGG |
| iNOS | GGAGCCTTTAGACCTCAACAGA | TGAACGAGGAGGGTGGTG |
| IL-10 | GCTCTTACTGACTGGCATGA | CGCAGCTCTAGGAGCATGTG |
| CD36 | GCTTGCAACTGTCAGCACAT | GCCTTGCTGTAGCCAAGAAC |
| FABP | CTGTGGAAAGGAAGCCTCGTT | CTCAAAGTTCTCCTGGCTCTGC |
| FASN | TGGGTTCTAGCCAGCAGAGT | ACCACCAGAGACCGTTATGC |
| SREBP-1c | CCTAACGTGGGCCTAGTCCGAAGCC | CCAGTTCGCACATCTCGGCCA |
| ACC1 | GGCTGGGCTTGTATAAGT | GATAATATGGCAATCTCAGT |
| ACOX | ACCAACTGTCACACTAACATATCA | GGCATGTAACCCGTAGCACT |
| CPT1 | ACATCCCTAAGCAGTGCCAG | TCGTCCGGCACTTCTTGATC |
| ATGL | CTTGAGCAGCTAGAACAATG | GGACACCTCAATAATGTTGGC |
| HGL | GCTGGAGGAGTGTTTTTTTGC | AGTTGAACCAAGCAGGTCACA |
| MGL | TCGGAACAAGTCGGAGGT | TCAGCAGCTGTATGCCAAAG |
| GAPDH | GCCAAACGGGTCATCATCTC | GTCATGAGCCCTTCCACAAT |
| **Human** |  |  |
| α-SMA | CGTGGGTGACGAAGCACAG | GGTGGGATGCTCTTCAGGG |
| Col1a1 | AAGAGTGGAGAGTACTGGATT | GTTCTTGCTGATGTACCAGT |
| FASN | TGCTCCCAGCTGCAGGC | GCCCGGTAGCTCTGGGTGTA |
| SREBP-1c | GGAGGGGTAGGGCCAACGGCCT | CATGTCTTCGAAAGTGCAATCC |
| CHOP | CTTGACCCTGCTTCTCTGGCTT | TTCCGTTTCCTGGTTCTCCCTT |
| CPT1 | CAATCGGACTCTGGAAACG | CCGCTGACCACGTTCTTC |
| IL-6 | GGATTCAATGAGGAGACTTGCC | TCTGCAGGAACTGGATCAGG |
| IL-1β | GGGACAGGATATGGAGCAACA | TTTCAACACGCAGGACAGGTA |
| MCP-1 | CCCCAGTCACCTGCTGTTAT | AGATCTCCTTGGCCACAATG |
| GAPDH | CCACTCCTCCACCTTTGAC | ACCCTGTTGCTGTAGCCA |
